# Safety and Use of MLC601/MLC901 (NeuroAiD^TM^) in Primary Intracerebral Hemorrhage: A Cohort Study from the NeuroAiD Safe Treatment Registry

**DOI:** 10.3390/brainsci10080499

**Published:** 2020-07-30

**Authors:** Ramesh Kumar, Azizi Abu Bakar, Jegan Thanabalan, Sanmugarajah Paramasvaran, Charng Jeng Toh, Ainul Jaffar, Farizal Fadzil, Palaniandy Kamalanathan, Bee Hong Soon, Narayanaswamy Venketasubramanian

**Affiliations:** 1 Department of Neurosurgery, Faculty of Medicine, Universiti Kebangsaan Malaysia Medical Centre, Kuala Lumpur 56000, Malaysia; abazizi@gmail.com (A.A.B.); jegan@ppukm.ukm.edu.my (J.T.); paramasvarans@yahoo.com (S.P.); cjtoh72@yahoo.com (C.J.T.); asjaafar@yahoo.co.uk (A.J.); nurelisha@yahoo.com (F.F.); pkpknathan@hotmail.com (P.K.); bsoon00@yahoo.com (B.H.S.); 2 Raffles Neuroscience Centre, Raffles Hospital, Singapore 188770, Singapore; drnvramani@gmail.com

**Keywords:** MLC601, MLC901, intracerebral hemorrhage, hemorrhagic stroke, clinical outcomes, registry

## Abstract

Background: MLC601/MLC901 (NeuroAiD™) is a combination of natural products shown to be safe and to aid neurological recovery after brain injuries, especially ischemic stroke. Few studies have investigated NeuroAiD in primary intracerebral hemorrhage (ICH). The NeuroAiD Safe Treatment (NeST) Registry explores NeuroAiD use in the real-world setting. This cohort study aimed to assess its use and safety in ICH. Methods: The online NeST Registry of subjects with ICH given NeuroAiD prospectively collected clinical data at baseline and monthly visits (V) 1 to 3. Outcome measures included compliance, side effects, Glasgow Coma Scale (GCS), National Institutes of Health Stroke Scale (NIHSS), modified Rankin Scale (mRS), and Short Orientation-Memory-Concentration Test (SOMCT). Results: Sixty-six subjects were included. NeuroAiD was well-tolerated with fair compliance over three months. Two non-serious side effects were reported. Mean scores significantly improved on all outcome scales. The proportion of subjects with favorable outcomes significantly improved from baseline to V3: NIHSS 0–4, from 12% to 59% (*p* < 0.0001); GCS 13–15, from 64% to 88% (*p* = 0.007); mRS 0–1, from 9% to 37% (*p* = 0.004); and SOMCT score 0–8, from 44% to 68% (*p* = 0.029). Conclusions: NeuroAiD in the real-world setting was safe and showed potential for a sustained positive effect on neurological recovery after ICH.

## 1. Introduction

Primary intracerebral hemorrhage (ICH) is the second most common subtype of stroke worldwide, accounting for more than 20% of all strokes [1]. Its global burden continues to increase. The highest incidence is among Asians, with low- and middle-income countries disproportionately affected. Compared with ischemic stroke, ICH has higher rates of mortality and leads to more severe disability. ICH is a medical emergency with a high rate of poor long-term outcomes [2]. Up to 40% of patients with ICH die within the first month, and only 12% to 39% of survivors achieve functional independence [3].

Following the bleeding event in ICH, a cascade of neurological injuries proceeds. The hematoma itself causes physical damage and mass effect on the surrounding brain tissue, which can lead to neuronal and glial ischemia, edema, and cell necrosis. Degradation byproducts from the hematoma cause the production of oxygen free radicals and release of pro-inflammatory signals, resulting in further injury. The recovery process involves the resolution of the clot and acute injury as well as neurorepair, including neurogenesis and neuronal network plasticity [4].

Clinical trials have failed to show improvement in functional outcomes using any specific therapy for ICH [5]. There is an urgent need for more translational research to develop therapies for lowering the risk of ICH occurrence, controlling bleeding in the hyperacute phase, managing the acute effects of ICH, facilitating recovery, and preventing ICH recurrence. The Hemorrhagic Stroke Academia Industry (HEADS) Roundtable in 2018 highlighted the challenges of conducting ICH trials, including the heterogeneity of the disease and difficulties in patient recruitment. The roundtable also called out the need for interventions aiding neurological recovery, as well as the importance of gathering evidence on the real-life use of specific ICH therapies [6].

MLC601/MLC901 (NeuroAiD™) is a product used as a natural medicinal intervention for enhancing recovery after neurological injury such as ischemic stroke and traumatic brain injury (TBI). MLC601 is a formulation consisting of nine herbal components (Radix *Astragali*, Radix *Salvia miltiorrhizae*, Radix *Paeoniae rubra*, *Rhizoma chuanxiong*, Radix *Angelicae sinensis*, *Prunus persica*, *Carthamus tinctorius*, Radix *Polygalae*, and *Rhizoma acori tatarinowii*) and five non-herbal components (*Cornu saigae tataricae*, *Buthus martensii*, *Hirudo*, *Eupolyphaga seu steleophaga*, and *Calculus bovis artifactus*). MLC901 is a simplified formulation containing only the herbal components [7]. Both formulations are pharmacologically equivalent and contain the molecules astragaloside IV, salvianolic acid B, and tanshinone IIB, which may amplify neurorepair processes. MLC601/MLC901 induces neurogenesis by stimulating the expression of brain-derived neurotrophic factor. This also promotes neurite outgrowth and synaptogenesis [8,9]. In regulating the neuroinflammation cascade, NeuroAiD modulates innate immunity by significantly reducing pro-inflammatory cytokines and chemokines [10]. Similarly, animal models in TBI demonstrated the neuroregenerative properties of NeuroAiD through the processes of neurogenesis, gliogenesis, and angiogenesis (via vascular endothelial growth factor) [11].

For post-stroke recovery, NeuroAiD is given for three months in addition to standard stroke care and rehabilitation. With long-term follow-up up to two years, NeuroAiD was shown to significantly improve the odds of achieving functional independence compared to placebo [12]. An even larger treatment effect is evident from the combination of NeuroAiD and persistent rehabilitation [13]. For cognitive outcomes after TBI, NeuroAiD given for six months showed significantly better improvement in complex attention and executive functioning compared to placebo [14]. Clinical trials have confirmed the long-term safety of NeuroAiD. Side effects are mostly mild, transient, and usually gastrointestinal (e.g., nausea, vomiting, and diarrhea).

Case reports and case series on NeuroAiD used in neurosurgical pathologies have been presented in international congresses [15,16]. Given the neurorestorative properties of NeuroAiD seen in ischemic stroke and TBI, NeuroAiD represents an attractive therapeutic option to address the neurological sequelae and to enhance neurorepair in the damaged neuronal environment in ICH and similarly in other brain injury pathologies.

While there are presently no published clinical trials on NeuroAiD in ICH, the NeuroAiD Safe Treatment (NeST) Registry was established to provide information on its use and safety in clinical practice [17]. Currently, the registry has voluntary participation from more than 500 subjects in over 10 countries. Among the indications of use in the registry, ICH ranks second to ischemic stroke in frequency. The present analysis aims to evaluate the use and safety of NeuroAiD in the real-world setting among subjects with ICH.

## 2. Materials and Methods

The ongoing NeST Registry (Clinicaltrials.gov Identifier NCT02536079) is designed as a product-specific prospective cohort study. Participation is entirely voluntary. The treating physician and the subject discuss the decision to use NeuroAiD before considering participation in NeST. Informed consent is obtained from all participants or their legal representatives. The study protocol was published in 2015 [17]. This analysis included a cohort of NeST subjects with ICH from Universiti Kebangsaan Malaysia (UKM) Medical Centre. NeST was approved by the UKM Research Ethics Committee in November 2014 (FF-2014–382).

The following are the criteria for participation in NeST:

### 2.1. Inclusion Criteria

1. Male or female;
2. Any age;
3. Any patient who is taking or has been prescribed NeuroAiD for any duration as judged by the physician and/or the participant;
4. Agrees to be included in the registry and allows retrieval and analysis of data in accordance with local requirements.

### 2.2. Exclusion Criteria

1. Unwillingness to participate;
2. Contraindication to NeuroAiD.

NeuroAiD was taken per the prescription of the treating physician or per the usual dosage for MLC601 (four capsules three times a day) or MLC901 (two capsules three times a day). The recommended treatment duration was three months.

NeuroAiD is available in capsule form. It can be administered orally, or its contents can be diluted in water to be passed through an enteral feeding tube. Other treatments and medical interventions prescribed by the treating physician were allowed; concomitant medications were recorded in the database.

Assessments were performed at baseline (before NeuroAiD intake) and at visits (V) 1, 2, and 3. The recommended visit intervals were monthly. Anonymized data were collected through an online data entry system (http://www.neuroaid.com/en/nest/) compliant with the Health Insurance Portability and Accountability Act of 1996. Clinical data collected include the following:

1. Demographics: date of birth, sex, ethnicity;
2. The main diagnosis (indication) for taking NeuroAiD and date of onset;
3. Other relevant medical conditions;
4. NeuroAiD date started and dose;
5. National Institutes of Health Stroke Scale (NIHSS) score;
6. Glasgow Coma Scale (GCS) score;
7. Modified Rankin Scale (mRS) score;
8. Short Orientation-Memory-Concentration Test (SOMCT) score;
9. Compliance with intake of NeuroAiD;
10. Occurrence of any side effects related to NeuroAiD: date of onset, date of resolution, and severity.

For this analysis, favorable outcomes were defined as follows: NIHSS score range of 0–4 (no or mild deficits); GCS score range of 13–15 (no or mild impairment of consciousness); mRS score range of 0–1 (functionally independent); SOMCT score range of 0–8 (within normal limits for cognition).

In this study, a side effect was defined as any unintended adverse event (AE) considered by the treating physician as possibly, probably, or definitely related to NeuroAiD use. AE was defined as any untoward medical occurrence in a person administered a product and which did not necessarily have a causal relationship with this treatment. An AE was considered serious if it resulted in death, a persistent or significant disability, abortion, congenital anomaly, or birth defect, was life-threatening, or required inpatient hospitalization or prolongation of existing hospitalization.

Descriptive statistics were used to summarize data. Cases of side effects reported in the registry were tabulated. Outcome assessments were compared to baseline and previous observations. The McNemar test was used to compare the proportion of subjects with favorable outcomes between baseline and each follow-up visit. For all outcome scales, the paired sample *t*-test or Wilcoxon signed-rank test was used to test if the change from baseline was significantly different from zero.

NeST is a proactive industry–academic collaboration supported by Moleac, the manufacturer of NeuroAiD. Central coordination (including administrative support, maintenance of the database and online data collection tool, data management, and preparation of periodic reports) is provided by the medical affairs and information technology departments of Moleac.

## 3. Results

This cohort included 66 subjects with ICH from Universiti Kebangsaan Malaysia Medical Centre who were consecutively entered in the registry. Data extraction was completed in February 2020. This registry-based study had variations in the maturity and completeness of data.

### 3.1. Baseline Characteristics

The median age was 58.5 (range 28–87) years. Females were 33%. Table 1 shows the baseline characteristics of this cohort. At baseline, median NIHSS was 10.5 (moderate severity) and median mRS was 4 (moderately severe disability). Median time from the onset of ICH to the first dose of NeuroAiD was 11 days.

### 3.2. Safety and Compliance

No serious side effects were reported. Side effects included a mild case of flushing and a moderate case of lip ulcer. No gastrointestinal side effects were noted. In accordance with inclusion criteria, all subjects were started on NeuroAiD and compliance to intake was fair over three months. By V3, more than half of the subjects reported intake to be either “often” or “always” as prescribed.

### 3.3. Clinical Outcomes

Outcome assessments at baseline and visits 1 to 3 are shown in Table 2. Mean scores on all outcome scales significantly improved from baseline to V3. The proportion of subjects with favorable outcomes on all scales significantly increased from baseline to V3.

**Figure 1:**
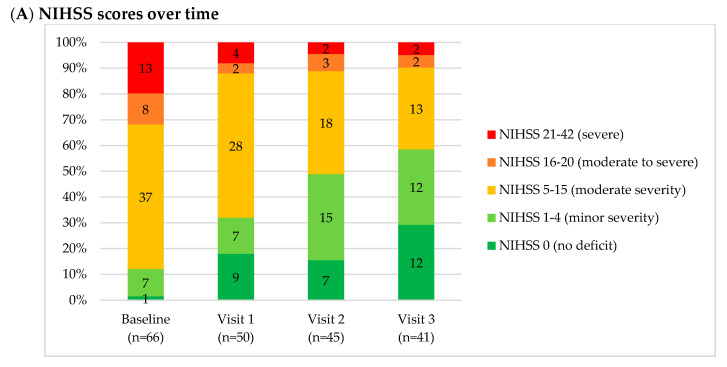

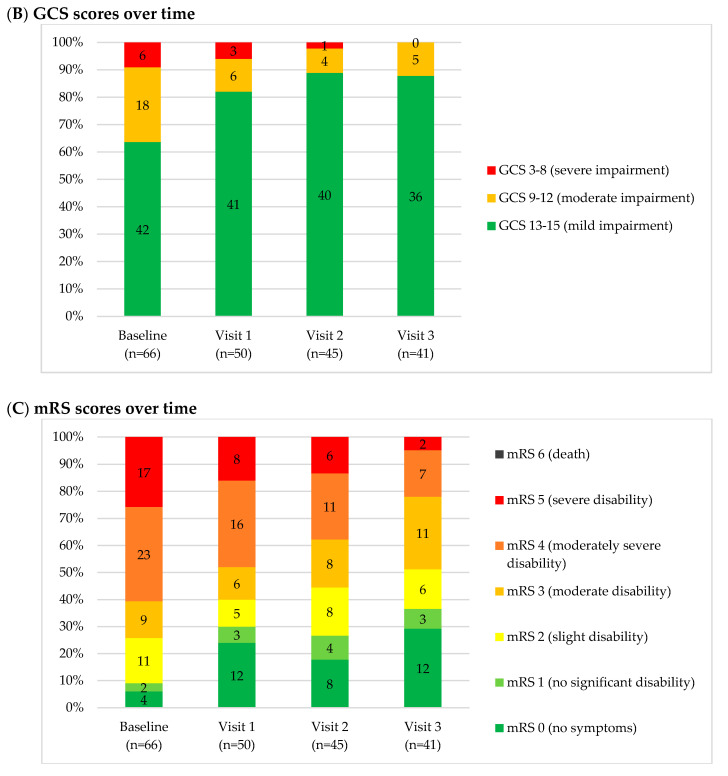

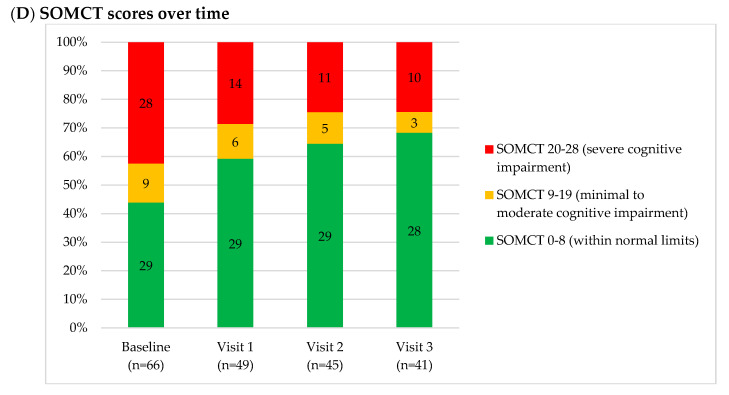
100% stacked bars. (**a**) National Institutes of Health Stroke Scale (NIHSS) scores over time. (**b**) Glasgow Coma Scale (GCS) scores over time. (**c**) modified Rankin Scale (mRS) scores over time. (**d**) Short Orientation-Memory-Concentration Test (SOMCT) over time. Indicated inside the bars is *n* for each category.

Neurological impairment from ICH significantly improved over time (Figure 1a). At baseline, only 12% of the subjects were NIHSS 0–4. By V3, the proportion increased almost five times to 59%. Impairment of consciousness from ICH significantly improved over time (Figure 1b). At baseline, only 64% of the subjects had GCS 13–15. By V3, the proportion increased to 88%. Functional independence improved over time (Figure 1c). At baseline, only 9% of the subjects had mRS 0–1. By V3, the proportion increased by more than four times to 37%. Cognitive impairment from ICH improved over time (Figure 1d). At baseline, only 44% had a SOMCT score of 0–8. By V3, the proportion increased to 68%.

## 4. Discussion

NeuroAiD had a favorable safety profile in this cohort of subjects with ICH followed up for three months in the real-world setting. The cohort experienced improvement in neurological, functional, and cognitive outcomes at three months compared to baseline. Given the current lack of well-designed clinical trials for specific ICH therapies and the challenges of their successful implementation, this registry-based prospective cohort study provides much-needed information on the real-world use and safety of NeuroAiD in ICH in clinical practice. Previous clinical trials of NeuroAiD explored its application in ischemic stroke and TBI, but not ICH.

Compared to registries, clinical trials have strict eligibility criteria with more restrictions on comorbid conditions and concomitant medications. Registries generate real-world evidence in the broader population in order to confirm and support efficacy and safety data gleaned from clinical trials. Furthermore, registries may detect signals of benefit and safety that could mitigate the risks of embarking prematurely on randomized controlled trials [18].

In this study, no mortality was reported. This may be due to a delay in the time from ICH onset to baseline assessment with a median of 12 days leading to selection bias. In addition, this cohort had minimal impairment of consciousness at baseline (median GCS of 15) but with moderate neurological deficits (median NIHSS of 10.5) and moderately severe disability (median mRS of 4). Nonetheless, no serious side effects were experienced by this cohort, confirming the well-established safety of NeuroAiD in prior clinical trials conducted in stroke and TBI [12,14]. Treatment was well-tolerated, with fair compliance over a period of three months.

In our registry-based cohort, the baseline NIHSS indicated moderate severity of neurological impairment, which is known to be a prognostic factor for poorer outcome [19]. Despite this, there was agreement across all outcome scales that this cohort experienced improvement over time until V3. The increase in the proportion of subjects with favorable outcome was most evident using NIHSS and mRS, which are the scales that have relatively more categories to demonstrate improvement compared to the scoring for GCS and SOMCT.

Favorable outcomes on GCS and SOMCT were already present at baseline in a relatively large proportion of subjects, 64% and 44% respectively. However, further improvements were still observed. In a Malaysian study describing clinical outcomes of ICH, the mean age (61.6 years) was similar to that of this study, but with more females (50%). Hypertension and diabetes were similarly prevalent. The reported mortality at 30 days was 43.9%. At one year, only 33.3% achieved good recovery [20].

This study has several limitations. Biases are difficult to control in registries. Subjects voluntarily participate and are aware of the treatment given. Subjects take NeuroAiD according to their best judgment, or that of their treating physician. Since this study reflects actual clinical use of NeuroAiD, there are variations among subjects in terms of maturity and completeness of the available data. There is no concurrent control arm for comparison. Treatment effect may be confounded by wide variabilities in baseline characteristics, concomitant medications, and other medical interventions. A well-designed randomized controlled trial and a longer duration of follow-up would be required to account for the heterogeneity of clinical characteristics in ICH and the potential of ICH subjects for spontaneous neurological recovery of varying degrees. Future studies may also collect more information regarding clinical characteristics relevant to ICH, including the size and location of bleeding, details of neurosurgical care, and treatment of elevated intracranial pressure. On the other hand, our findings from this study describing the use and safety of NeuroAiD will be relevant in the design of future double-blind placebo-controlled trials to evaluate the efficacy and safety of NeuroAiD in ICH.

## 5. Conclusions

This registry-based cohort of ICH subjects followed up for three months confirms that NeuroAiD has a favorable safety profile and is well-tolerated with fair compliance. With NeuroAiD use in the real-world setting, this cohort experienced improvement for three months in neurological, functional, and cognitive outcomes. The efficacy and safety of NeuroAiD in ICH should be evaluated with double-blind placebo-controlled trials with a longer duration of follow-up.

## Figures and Tables

**Table 1:** Baseline characteristics.

Variable	Result (*n* = 66)
Age in years (Median, Range)	58.5 (28–87)
Male (*n*, %)	44 (67%)
Female (*n*, %)	22 (33%)
Most common comorbid conditions (*n*, %)	
Hypertension	55 (83%)
Diabetes	14 (21%)
Hyperlipidemia	11 (17%)
Time from ICH onset to baseline assessment, in days (Median, Range)	12 (0–238)
Time from ICH onset to first NeuroAiD dose, in days (Median, Range)	11 (0–238)
Outcome measure (Median, Range)	
NIHSS score	10.5 (0–33)
GCS score	15 (3–15)
mRS score	4 (0–5)
SOMCT score	14 (0–28)

NIHSS, National Institutes of Health Stroke Scale; GCS, Glasgow Coma Scale; mRS, modified Rankin Scale; SOMCT, Short Orientation-Memory-Concentration Test.

**Table 2:** Outcome assessments at baseline and visits 1 to 3.

Assessment	Baseline (*n* = 66)	Visit 1 (*n* = 50)	Visit 2 (*n* = 45)	Visit 3 (*n* = 41)	*p*-Value ^#^
NIHSS					
Mean (SD)	12.7 (8.3)	8.1 (7.5)	6.8 (7.6)	5.2 (6.6)	<0.0001 *
Median (Q1, Q3)	10.5 (6, 19)	7 (2, 11)	5 (2, 9)	4 (0, 7)	
Min, Max	0, 33	0, 33	0, 34	0, 31	
Score 0–4, *n* (%)	8 (12.1)	16 (32)	22 (48.9)	24 (58.5)	<0.0001 ^$^
GCS					
Mean (SD)	12.8 (3.1)	13.9 (2.5)	14.3 (2.1)	14.5 (1.3)	0.001 **
Median (Q1, Q3)	15 (11, 15)	15 (15, 15)	15 (15, 15)	15 (15, 15)	
Min, Max	3, 15	3, 15	3, 15	10, 15	
Score 13–15, *n* (%)	42 (63.6)	41 (82.0)	40 (88.9)	36 (87.8)	0.007 ^$^
mRS					
Mean (SD)	3.5 (1.4)	2.7 (1.9)	2.6 (1.7)	2.1 (1.6)	<0.0001 **
Median (Q1, Q3)	4 (2, 5)	3 (1, 4)	3 (1, 4)	2 (0, 3)	
Min, Max	0, 5	0, 5	0, 5	0, 5	
Score 0–1, *n* (%)	6 (9.1)	15 (30.0)	12 (26.7)	15 (36.6)	0.004 ^$^
SOMCT					
Mean (SD)	13.8 (12.5)	10.5 (11.2)	8.6 (11.0)	8.4 (11.6)	0.032 **
Median (Q1, Q3)	14 (0, 28)	6 (0, 22)	2 (0, 16)	0 (0, 16)	
Min, Max	0, 28	0, 28	0, 28	0, 28	
Score 0–8, *n* (%)	29 (43.9)	29 (59.2)	29 (64.4)	28 (68.3)	0.029 ^$^

NIHSS, National Institutes of Health Stroke Scale; GCS, Glasgow Coma Scale; mRS, modified Rankin Scale; SOMCT, Short Orientation-Memory-Concentration Test; ^#^ Visit 3 versus baseline; * paired sample *t*-test; ** Wilcoxon signed-rank test; ^$^ McNemar’s test.
